# Emerging Technologies for Cancer Prevention and Other Population Health Challenges

**DOI:** 10.2196/jmir.7.3.e30

**Published:** 2005-07-01

**Authors:** Thomas R Eng

**Affiliations:** ^1^eHealth Institute

**Keywords:** Public health, public health informatics, population surveillance, cancer, prevention

## Abstract

Emerging technologies, such as information and communication technologies (including future versions of the Internet), microelectromechanical systems, nanotechnologies, genomics, robotics, artificial intelligence, and sensors, provide enormous opportunities for enhancing health and quality of life. Population health technologies (PHTs) encompass the various applications of emerging technologies to improve the health of populations and communities. These technologies may change many population health paradigms, including those related to cancer prevention and control. In the future, emerging technologies will allow true customization of health communication to individuals, and existing tailoring approaches will be considered very crude. Environmental monitoring systems based on emerging technologies could also provide real-time information that health officials and community residents could use immediately to ameliorate potential carcinogenic or unhealthy exposures. Accelerating the application and diffusion of emerging technologies to population health challenges will require a multipronged approach, including new transdisciplinary programs, increased funding, supportive infrastructure, and policy changes.

## Emerging Technologies and Health

Technological advances, such as pasteurization, sanitation, childhood immunization, food fortification, and car safety belts, have contributed substantially to the reduction of mortality and morbidity during the last two centuries. During the past several decades, the pace of technological innovation and discovery has been exponential. For example, when the first mainframe computer was built in the 1940s, it weighed more than 30 tons and occupied a room the size of a house. In 2002, standard microprocessors found in personal computers were more than 100000 times more powerful, and their weight is measured in grams. Years from now, DNA-based computers may be many times faster than today's advanced supercomputers, and their weight will be measured in nanograms [1].

At the beginning of the twenty-first century, emerging technologies provide enormous opportunities for further improvements in health and quality of life. These emerging technologies are being applied to many areas in medicine, including cancer diagnosis and treatment, where they are being deployed in applications such as detection of early cancer precursors, minimally invasive surgery, and molecular level diagnosis and treatment [2,3]. Recent cancer-related technologies have had considerable impact on cancer care and survival. As cancer care technology advances, it is possible that many cancers will eventually be viewed as chronic diseases.

Whereas there is substantial research, development, and investment in advancing the use of emerging technologies in biomedical interventions such as diagnostics and treatments, there is considerably less funding and interest in applying new technologies to population-oriented interventions. The application of emerging technologies to population health problems represents an exciting opportunity to address long-standing population health problems related to cancer prevention and control.

## Population Health Technology

Population health technologies (PHTs) encompass the various applications of emerging technologies to improve the health of populations and communities [4]. Examples of emerging technologies that have direct applications to population health include information and communication technologies (including future versions of the Internet), microelectromechanical systems, nanotechnologies, genomics, robotics, artificial intelligence, and sensors [5,6].

A population health model focuses on issues and interventions that impact populations and communities rather than individuals. It emphasizes prevention and focuses on those eHealth technologies that improve health on a population level rather than in an individually focused, medical care context. Thus, PHTs tend to include preventative, behavioral, environmental, social, and systems-oriented technologies rather than biomedical ones, such as diagnostics and treatment modalities.

The core principles of PHTs include a collaborative, multidisciplinary approach to development of health interventions. Relevant disciplines include the biological, physical, and social sciences; engineering; health care; public health; and business. Although PHTs by definition employ leading edge technology, the technology is often as transparent as possible as the developers focus on people and processes rather than on the technology.

Potential health issues that could benefit from the use of emerging technologies include the following:

- Disease (health) surveillance and control
- Environmental monitoring and pollution prevention
- Food and water safety
- Health communication and behavior change
- Self-care and chronic disease management
- Population screening
- Injury prevention and control
- Wellness and social isolation
- Health disparities

Given the great spectrum of possible technological solutions for population health, a comprehensive discussion of PHTs is not possible in this paper. Instead, two of the more compelling potential PHT applications related to cancer prevention and control—tailored health communication and environmental monitoring—are highlighted to illustrate the potential impact of PHTs. Given that PHT is an emerging field, there are limited data, scientific literature, and project experiences to support some of the concepts in this paper.

## Tailored Health Communication

Some eHealth developers vary the content, presentation, and/or medium of health content to an individual user based on knowledge about that individual. The expectation is that a tailored message or experience engineered to appeal to a specific individual is more likely to move the user along the stages of the change continuum compared to a generic message or experience [7].

Tailored online communication is typically based on a limited set of variables that are thought to influence the individual's receptiveness, comprehension, and perceived relevancy of the message. Currently, tailoring approaches include those based on user demographics, self-reported preferences, and usage of a website or technology. Technically, most current tailoring approaches are really not “tailored,” but rather, they are based on gross generalizations about heterogeneous groups of people.

The most common set of tailoring variables used for online communication seems to be demographic attributes. Examples of this include the segmentation of Web pages and, in some cases, entire Web domains, into age (eg, children, teens, and seniors), gender, and racial/ethnic groups (eg, African, Asian, Hispanic, and Native American). Another commonly used approach to online tailoring is to vary messages depending on self-reported preferences. Individual preferences may include specific forms of media (eg, text, audio, or video), user interfaces or display formats (eg, personal computer, PDA, or wireless phone), or reading level. Such preferences may be collected through brief one-time online questionnaires presented to users or through user registration forms for those who want access to additional functionality or content as a registered user.

A less common but emerging approach to online tailoring is to vary messages based on an individual's use of a website or technology. Software technology, usually in the form of a “cookie,” is used to track viewed pages and other movements of an individual within or across websites. Specific pages are presented to the user based on assumptions that depend on his or her usage patterns. Such assumptions may be based on simplistic deductions about the user (eg, if someone clicks on a hyperlink to a page about cancer among women, then the user is probably a woman), or they may be based on fairly complex algorithms. To the author's knowledge, few if any widely used health websites employ the latter technique.

In the future, emerging technologies will allow true tailoring of communication to specific individuals, and existing personalization approaches will be considered very crude. The advent of sophisticated devices and systems for collecting, transmitting, and interpreting data generated by individuals and the environment may serve as the nidus for the development of tailoring algorithms that may surpass our current abilities to match messages with users. Not only will we be able to better match messages to the individual, but we will be able to match versions of such messages in the context of the user's microenvironment at the time of decision making. This is because specific versions of a message may be more appropriate for certain decision-making contexts than others. In addition, we may be able to create “dynamic” messages, which can adapt themselves depending on minute changes in the user's microenvironment. Thus, the permutations of possible messages and their presentations to the individual could be in the millions as opposed to the dozens many online communicators now employ.

It is possible that future tailoring schemas will be based on classes of variables that describe individual attributes that have not been accounted for by current developers, including the following:

- Who you are – motivations, personality profile
- What you have experienced – social, health, and medical history
- What you are – genetics, physiological profile, medications
- Where you are – physical setting, microenvironment, point in the decision-making continuum
- How you are – physical and mental status, mood

The technologies required in order to implement the advanced tailoring approaches described above include ubiquitous electronic health information systems and devices that collect data from both health care and non-health care settings. The huge volume of data generated by these emerging devices means that robust data storage and transmission infrastructures are needed. And, in order to provide “just-in-time” personalization, sensors will need to be developed that can capture information about the individual's microenvironment at the time of decision making. Sophisticated algorithms will also be needed to interpret multiple streams of data from sensors in order to accurately describe changes in the microenvironment.

## Environmental Monitoring

Many types of cancer are associated with exposure to environmental toxins. These toxic substances may be found in air, water, food, and soil. Lifestyle and work choices are important determinants of exposure to environmental toxins.

 Various governmental jurisdictions have formal programs to monitor air, water, food, and soil for known environmental toxins. However, despite recent attempts to update such systems, most environmental monitoring systems have substantial shortcomings. For example, in the case of ambient air monitoring, only a small number of pollutants is tested and only periodic testing is conducted [8]. In addition, air sampling stations are usually placed high on buildings rather than at the level where people typically breathe. As a result, the data generated by current monitoring programs are only representative of a small number of locations at limited points in time. They typically are not representative of the microenvironments experienced by individuals during their daily activities. And, because reporting of most monitoring data is delayed and not available in real time, the data are not actionable and are relatively inaccessible to the people who are the ultimate users of the data.

Emerging technologies may be applicable in developing environmental monitoring systems that can provide accurate and timely assessments of environmental health hazards. Monitoring systems based on emerging technologies could provide real-time information that health officials and residents could use immediately to ameliorate potential carcinogenic or unhealthy exposures. Providing real-time, continuous information about the air that actually surrounds individuals during daily activities would offer a more accurate and representative picture of the public's exposure to toxins.

There are several possible models for real-time, representative air pollution monitoring systems, all of which would require the enhancement of existing technologies. One possible system would consist of representative individuals (ie, citizen sentinels) who volunteer to wear a sensor during their daily activities. The wearable sensor would sample small amounts of air and analyze them for specific pollutants many times an hour or continuously. The data would be transmitted wirelessly in real time to central servers. These servers would then use complex algorithms to analyze and interpret the data for health officials and the public. Finally, easy to understand interpretations of the data and action-oriented messages (eg, “unhealthy air—limit outdoor activity now”) for the public could be shown on public displays (akin to highway message signs) or sent to subscribers to their preferred messaging device. Such a system would allow the public to take appropriate action to limit exposure to pollutants, which should be the primary objective of pollution monitoring systems.

## Cautionary Factors

As we move forward in developing and deploying PHTs, developers and policy makers should address the following issues to ensure that these products actually benefit public health and do not have unintended consequences.

### Privacy, Confidentiality, and Security

Many PHTs, especially those related to cancer prevention and control, will collect, analyze, and transmit sensitive health information. The ability of developers to balance public concerns about privacy with the data needs of PHTs will be an important determinant of success. Government regulations are typically behind the pace of technological innovation and are often not responsive to cutting-edge technologies or business models [9]. Thus, robust policies, voluntary or otherwise, will be needed to comprehensively address the upcoming exponential growth of health data generated by networked devices, such as information appliances and sensors. Failing to address the public's concerns about privacy, confidentiality, and security would jeopardize the widespread adoption of many PHTs.

### Unintended Effects and Quality and Effectiveness

Rigorous outcome studies of PHT products are limited because these products have not been widely deployed. Given that many PHTs, by definition, will use technologies that have not been used widely in the marketplace, the potential for unintended errors and ineffective products is real. In addition, it is possible that some emerging technologies, such as nanotechnologies, may have deleterious health effects [10]. When possible, PHT developers should consider the evidence base for their technologies, integrate quality improvement and evaluation processes into the product development lifecycle, and build evaluation components into their product development and implementation plans [11,12].

### Sustainability

There are legitimate concerns about the sustainability of many PHT and other eHealth products [13]. Because many PHTs do not have precedents, the strength of market demand for these technologies is largely unknowable until they are introduced to consumers and consumers are educated about the benefits of such technologies. Public funds have traditionally been the primary source of support for population health programs, but other possible sources of support, including end users and health intermediaries (such as corporations, employers, health care providers, and health plans), should be explored. Given the uncertainly of funding, PHT developers will need to examine new business models for sustaining PHTs.

### Technological Divide

As the field moves forward, developers and policy makers will need to ensure equal access to technologies that improve population health [14]. One approach may include subsidizing the use of PHTs among underserved populations from a portion of the proceeds of sales to organizations with greater resources. It is likely, however, that some type of government or foundation support for the use of PHTs among certain underserved populations will be needed.

## Moving the Field Forward

Although some PHTs have begun to emerge in response to the recent threat of bioterrorism, emerging technologies are rarely being applied to population health problems. The author is not aware of any formal public or private programs that explicitly fund development and dissemination of PHTs.

Reasons for the lack of focus on PHTs include the following:

- There is a lack of national and global leadership and infrastructure to promote and support the development and dissemination of PHTs. Some government programs support technology research and development in specific interest areas, but none focus on population health.
- Most research, development, and investment activities related to emerging technologies focus on individually oriented medical care interventions (eg, pharmaceuticals, medical devices, diagnostics) rather than on population health opportunities.
- Development of PHTs requires a multidisciplinary and multisector approach involving stakeholders who do not usually communicate or collaborate with each other.
- Public health institutions have not been successful in technology transfer and commercialization of innovations primarily because they often lack the entrepreneurial capacity or market understanding to transform technological concepts into viable products.
- There is a lack of professional and public understanding of PHTs.

Accelerating the application and diffusion of emerging technologies to population health challenges will require a multipronged approach. Several key areas will need to be addressed to lay the foundation for this new field of endeavor.

### Promoting Transdisciplinary Approaches

Because technologies with population health applications will likely originate from a variety of sectors, such as computer science, health care, public health, genomics, nanotechnology, environmental science, and engineering, networks of individuals and organizations in these disciplines will need to be created. The silo nature of health professional and technology education at universities should be re-examined to see how students can concurrently develop skills and experience in multiple areas, including population health, technology development, and business. In addition, more networking opportunities for professionals, such as the annual eHealth Developers' Summit, that foster business relationships and collaboration among health technology developers and funders from commercial entities, academia, government, and nonprofits are needed [15].

### Increasing Funding

Given the high risk but high societal impact of most PHTs, government agencies and private foundations should consider more funding for PHT research, development, and dissemination. Private investors will need to be educated about the market opportunities around these technologies in order to encourage more private sector investment.

### Developing Infrastructure

National and global infrastructures need to be enhanced to support PHT development and adoption, especially in underserved areas. Government initiatives, such as the National Health Information Infrastructure, should more explicitly support the development of infrastructure to enhance population health—not just for patient safety, health care quality, and bioterrorism prevention [16]. Supportive programs to help PHT developers produce viable products in the marketplace are also needed.

### Changing Policy

Potential policy changes that could promote widespread adoption of PHTs include reimbursement for effective technologies, realignment of incentives to reward quality and positive health outcomes, incentives for consumers to make healthy decisions, and redefinition of the roles and responsibilities of health professionals and institutions.

PHTs have the potential to positively change many paradigms in cancer prevention and control and other population health areas. With these technologies, it may be possible to cost-effectively screen entire at-risk populations for dozens of cancers and cancer precursors with a single drop of body fluid. It may be possible to detect individual and group exposures to carcinogens early enough to prevent disease. In addition, imagine being able to empower people to make the best health decisions at the exact time of decision making, and to enable communities to monitor and address local health and environmental issues before they become significant health hazards.

Transdisciplinary programs, increased explicit funding, supportive infrastructure, and policy changes will help accelerate the development and availability of a new breed of technologies that are likely to have substantial impacts on cancer prevention and other population health challenges.

## Abbreviations

PHTs: population health technologies
